# Neue Ansätze in der Immuntherapie gastrointestinaler Tumoren: Blick in die Glaskugel

**DOI:** 10.1007/s11377-025-00895-y

**Published:** 2025-04-04

**Authors:** Bernd Heinrich, Tim F. Greten

**Affiliations:** 1 Klinik für Gastroenterologie, Hepatologie, Infektiologie und Endokrinologe, Medizinische Hochschule Hannover, Carl-Neuberg-Str. 1, 30625 Hannover, Deutschland; 2 Thoracic and Gastrointestinal Malignancies Branch, Center for Cancer Research, National Cancer Institute, National Institutes of Health, 20892 Bethesda, MD USA; 3 Liver Cancer Program, Center for Cancer Research, National Cancer Institute, National Institutes of Health, Bethesda, USA

**Keywords:** Immuncheckpointinhibitor, Immunkonjugate, Adoptive Immuntherapie, Onkolytische Viren, Biomarker, Immune checkpoint inhibitor, Immunoconjugates, Adoptive immunotherapy, Oncolytic viruses, Biomarkers

## Abstract

Die Immuntherapie ist Teil der Standardtherapie von gastrointestinalen (GI‑)Tumoren. Dennoch sind Ansprechraten eher gering. Aktuelle Studien untersuchen den optimalen Zeitpunkt und die Patientenklientel für eine Immuntherapie. Auch die Kombinationen von zugelassenen Medikamenten werden getestet. Die Entwicklung neuer Therapieansätze ist ebenso wichtig um z. B. primäre und sekundäre Resistenzen überwinden zu können. Die antikörpervermittelte Immuncheckpointinhibitor(ICI)-Therapie wird stetig erweitert. Neue Zielmoleküle auf Immun- und Tumorzellen sollen eine weitere Verbesserung der Immunantwort durch Aktivierung von Immunzellen oder Blockade eines hemmenden Signalwegs erzeugen. Die Kombination aus Antikörper mit Arzneistoff im Sinne eines Immunkonjugats ist möglich. Modifikationen der Antikörperstruktur werden auf verbesserte Wirksamkeit und ein erweitertes Einsatzspektrum getestet. Zelluläre Strategien, wie der adoptive Zelltransfer oder die Applikation von gentechnisch veränderten T‑Zellen, werden aktuell in Studien für den Einsatz bei GI-Tumoren überprüft. T‑Zellen mit chimären Antigenrezeptoren (CAR), die bestimmte Proteine auf Tumorzellen erkennen und angreifen, sind ein vielversprechender Ansatz. Viren, die aufgrund des natürlichen Reproduktionsverhaltens oder genetischer Veränderungen Tumorzellen zerstören können, werden als onkolytische Viren in der GI-Onkologie eingesetzt, bedingen jedoch Herausforderungen durch geringe Immunogenität oder unspezifische Wirkung. Eine weitere Schwierigkeit ist die Entwicklung sensitiver und spezifischer Biomarker, die Ansprechen und Wirksamkeit von Immuntherapien voraussagen. Dieser Übersichtsartikel soll einen Blick in die Glaskugel erlauben und neue vielversprechende immuntherapeutische Ansätze präsentieren und diskutieren.

## Abstract (en)

Immunotherapy is part of the standard therapy for gastrointestinal (GI) tumors. Nevertheless, response rates are rather low. Current studies are investigating the optimal timing and patient clientele for immunotherapy. Combinations of approved drugs are also being tested. The development of new therapeutic approaches is equally important, e.g. to overcome primary and secondary resistance. Antibody-mediated immune checkpoint inhibitor (ICI) therapy is constantly being expanded. New target molecules on immune and tumor cells are intended to further improve the immune response by activating immune cells or blocking an inhibitory signaling pathway. The combination of antibody with drug in the sense of an immunoconjugate is possible. Modifications of the antibody structure are being tested for improved efficacy and an extended range of applications. Cellular strategies, such as adoptive cell transfer or the application of genetically modified T cells, are currently being tested in studies for use in GI tumors. T cells with chimeric antigen receptors (CAR), which recognize and attack certain proteins on tumor cells, are a promising approach. Viruses that can destroy tumor cells due to natural reproductive behavior or genetic alterations are used as oncolytic viruses in GI oncology, but pose challenges due to low immunogenicity or non-specific effects. Another difficulty is the development of sensitive and specific biomarkers that predict response and efficacy of immunotherapies. This review article aims to provide a glimpse into the crystal ball and present and discuss new promising immunotherapeutic approaches.

## Hintergrund

**Abb. 1:**
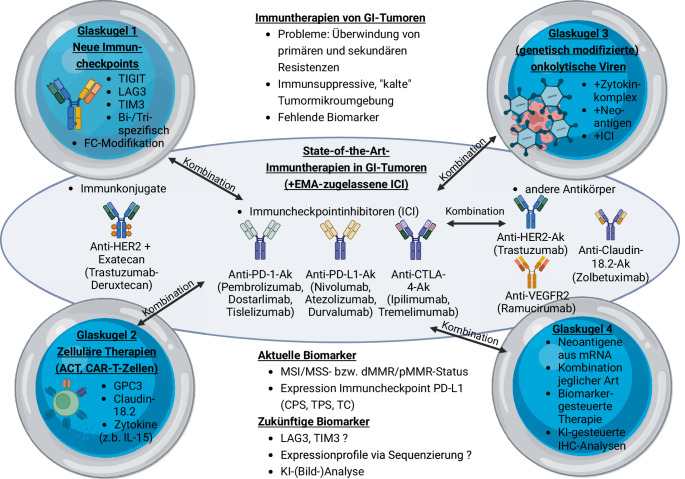
Aktuelle Immuntherapien bei gastrointestinalen (*GI-*)Tumoren sowie vielversprechende neue Therapiestrategien in der Glaskugel. *Ak* Antikörper, *ACT* adoptiver Zelltransfer, *CAR* chimärer Antigenrezeptor, *CPS* „combined positive score“, *dMMR* defizientes Mismatch-Reparatur-System, *EMA* Europäische Arzneimittelagentur, *GPC3* Glypican 3, *ICH* Immunhistochemie, *ICI* Immuncheckpointinhibitor, *IL* Interleukin, *KI* künstliche Intelligenz, *LAG3* „lymphocyte activation gene 3“, *MSI* Mikrosatelliteninstabilität, *MSS* Mikrosatellitenstabilität,* pMMR* kompetentes Mismatch-Reparatur-System, *TC* Tumorzellscore,* TIGIT* „T cell immunoreceptor with Ig and immunoreceptor tyrosine-based inhibitory motif (ITIM) domains“, *TIM‑3* „T cell immunoglobulin and mucin-domain containing 3“, *TPS* „tumor proportion score“. (Hergestellt mit BioRender, Toronto, Kanada)

Die Immuntherapie hat in den letzten Jahren signifikante Fortschritte in der Behandlung gastrointestinaler (GI-)Tumoren erzielt und stellt eine vielversprechende therapeutische Option dar. Die Ansprechraten und der Effekt auf das Langzeitüberleben bei GI-Tumoren ist dennoch optimierungsbedürftig. Die Immunaktivität in der Tumormikroumgebung spielt eine wichtige Rolle für die Wirksamkeit einer Immuntherapie. Neue immuntherapeutische Strategien versuchen die antitumorale Wirkung maximal zu steigern und primäre als auch sekundäre Resistenzen zu überwinden (Abb. 1).

Immunkonjugate zeigen Erfolge

Antikörpervermittelte Therapien und insbesondere Immuncheckpointinhibitoren (ICI) sind bereits Teil vieler leitliniengestützter Therapiealgorithmen. Die Entdeckung der Immuncheckpoints „programmed cell death protein 1“ (PD-1) bzw. dessen Liganden „programmed cell death protein ligand 1“ (PD-L1) und „cytotoxic T lymphocyte antigen 4“ (CTLA-4) und die damit einhergehende signifikante Veränderung der onkologischen Therapielandschaft wurde im Jahr 2018 mit dem Nobelpreis geehrt [42].

Die Behandlung von GI-Tumoren profitiert von weiteren Therapieoptionen aus dem Bereich der Immuntherapie. Die antikörpervermittelte Therapie wird stetig erweitert, da zusätzliche aktivierende oder hemmende Zielstrukturen auf Immun- und Tumorzellen, wie z. B. „T cell immunoreceptor with Ig and immunoreceptor tyrosine-based inhibitory motif (ITIM) domains“ (TIGIT), „lymphocyte activation gene 3“ (LAG3) oder „T cell immunoglobulin and mucin-domain containing 3“ (TIM-3), identifiziert und deren Signalwege blockiert werden können. Die Kombination aus einem Antikörper mit anderen Arzneistoffen im Sinne eines Immunkonjugats zeigt ebenso Erfolge. Ferner gehen molekulare Modifikationen der Antikörperstruktur mit verbesserter Wirksamkeit und erweitertem Einsatzspektrum einher.

Zellulär vermittelte Strategien werden aktuell in zahlreichen Studien auch bei GI-Tumoren überprüft. T‑Zellen mit chimären Antigenrezeptoren (CAR) sind dabei einer der vielversprechendsten Ansätze. Sie sind genetisch so modifiziert, dass sie bestimmte Proteine auf Tumorzellen erkennen und diese angreifen können.

Viren, die aufgrund ihres natürlichen Reproduktionsverhaltens oder genetischer Veränderungen Tumorzellen zerstören, werden als onkolytische Viren ebenso in der GI-Onkologie untersucht. Die Ansprechraten und Wirksamkeit in klinischen Studien sind noch gering und es bedarf weiterer Entwicklung.

Eine zusätzliche Herausforderung besteht darin, sensitive und spezifische Biomarker zu finden, die ein Ansprechen und die Wirksamkeit von Immuntherapien zuverlässig voraussagen können.

Dieser Übersichtsartikel soll einen Blick in die Glaskugel erlauben und neue vielversprechende immuntherapeutische Ansätze präsentieren und diskutieren. Dabei sollen innovative Entwicklungen der zuvor genannten Ansätze aufgeführt und in die aktuellen Therapieempfehlungen eingeordnet werden.

Um zukünftige Entwicklungen von Immuntherapien besser beurteilen zu können, soll ein kurzer Überblick über die aktuellen klinischen Standards für den Einsatz immuntherapeutischer Mittel bei soliden Tumoren des Gastrointestinaltrakts in Deutschland gegeben werden.

## Nachweis von Immuncheckpointmolekülen als Biomarker zur Therapiesteuerung

Der Nachweis von Immuncheckpointmolekülen auf Tumor- oder Immunzellen hat sich als guter Biomarker für die Steuerung von ICI-Therapien erwiesen. Die folgenden Klassifikationssysteme finden sich in aktuellen Leitlinien. Der „combined positive score“ (CPS) ist definiert als die Anzahl PD-L1-positiver Zellen (Tumorzellen, Lymphozyten, Makrophagen) bezogen auf die Gesamtzahl an Tumorzellen multipliziert mit 100. Der Tumorzellscore (TC) oder „tumor proportion score“ (TPS) gibt das prozentuale Verhältnis der PD-L1-positiven Tumorzellen zu allen vitalen Tumorzellen an.

## Mikrosatellitenstatus und tumoragnostischer ICI-Einsatz

Der Nachweis einer Mikrosatelliteninstabilität (MSI oder „MSI- high“ [MSI-H] ) bzw. defizientem DNA-Mismatch-Reparatur-System (dMMR) in Tumorzellen bedingt eine besonders hohe Wahrscheinlichkeit für die Entstehung von neuen immunogen wirkenden Proteinen (Neoantigene). Daraus folgt, dass MSI/dMMR-Tumoren ein ein gutes Ansprechen auf eine Immuntherapie zeigen. Tumoren mit Mikrosatellitenstabilität(MSS)- bzw. kompetentem DNA-Mismatch-Reparatur-System (pMMR-)Status oder ohne starke Expression von Checkpointmolekülen stellen weiterhin schwierig zu therapierende Subgruppen dar, für die aktuell keine guten immuntherapeutischen Optionen zur Verfügung stehen.

MSI/dMMR-Tumoren zeigen ein gutes Ansprechen auf eine Immuntherapie

Die Therapie mit Pembrolizumab ist bei vorbehandelten MSI-H/dMMR-Tumoren unabhängig von der Tumorentität zugelassen. Diese „tumoragnostische“ Therapie ist eine Besonderheit in der Onkologie und zeigt den Stellenwert der ICI. Die hohe Mutationsrate in MSI-H/dMMR-Tumoren sorgt für ein überdurchschnittliches Ansprechen der Immuntherapie, das in den Studien bei den 15 untersuchten Tumorentitäten im Schnitt bei 40 % lag [20].

In der GI-Onkologie ergeben sich dadurch folgende Zulassungen für Pembrolizumab bei Nachweis eines MSI-high/dMMR-Status des Tumors: So ist im Adenokarzinom des Ösophagus und Magens und im cholangiozellulären Karzinom in der Zweitlinientherapie die Monotherapie mit Pembrolizumab zugelassen (CheckMate-649; [15]; Keynote-859 [30]; Keynote-158; [23]). Am besten untersucht sind ICI bei MSI-H/dMMR-Kolonkarzinomen. Auch hier ist im metastasierten Stadium Pembrolizumab zugelassen, weitere Details im Folgenden unter *Kolon- und Rektumkarzinom*.

## State-of-the-Art-Therapie

### Ösophagus- und Magenkarzinom

Sowohl für das Adenokarzinom des Ösophagus, des ösophagogastralen Übergangs (AEG) und des Magens als auch das Plattenepithelkarzinom des Ösophagus werden Immuntherapien in der Standardtherapie eingesetzt. Dabei stellen diese Tumorentitäten ein Beispiel für den biomarkerbasierten Einsatz von Immuntherapien aufgrund der immunhistologischen Expression entsprechender Zielmoleküle dar.

Im fortgeschrittenen Adenokarzinom wird der Einsatz der Checkpointinhibitoren Nivolumab (Anti-PD-L1-Antikörper) oder Pembrolizumab (Anti-PD-1-Antikörper) in Kombination mit Chemotherapie als Erstlinientherapie empfohlen [38]. Die Empfehlung richtet sich nach dem CPS. Für das Ösophaguskarzinom ist Nivolumab bei einem CPS von ≥ 5 (gemäß CheckMate-649-Studie [15]) und Pembrolizumab bei einem CPS ≥ 10 (gemäß Keynote-590-Studie [37]) zugelassen. Für das Magenkarzinom und den AEG-Tumor besteht eine Zulassung für Pembrolizumab bereits bei einem CPS ≥ 1.

Weiterhin richtet sich die Therapie nach dem Status des „human epidermal growth factor receptor“ 2 (HER2), wobei der Antikörper Trastuzumab zusätzlich für HER2-positive Tumoren verwendet werden kann (Keynote 859 und Keynote 811 [14, 30]).

Die antikörpervermittelte Behandlung wird auch in der Zweit- und Drittlinientherapie eingesetzt

Die antikörpervermittelte Behandlung wird auch in der Zweit- und Drittlinientherapie eingesetzt. Es besteht die Zulassung für das Immunkonjugat Trastuzumab-Deruxtecan, ein Arzneistoff, bei dem an Trastuzumab das zytostatisch wirkende Exatecan gebunden ist [40].

Das Transmembranprotein Claudin-18.2 ist an der Zelladhäsion beteiligt. Der Antikörper Zolbetuximab richtet sich gegen Claudin-18.2 und ist in Kombination mit Chemotherapie für Magenkarzinome zugelassen, die dieses Molekül vermehrt exprimieren [34].

Im Plattenepithelkarzinom wird nach Radiochemotherapie und chirurgischer Resektion der Einsatz von Nivolumab als adjuvante Therapiestrategie gemäß CheckMate-577-Studie empfohlen [17]. Beim fortgeschrittenen Plattenepithelkarzinom des Ösophagus besteht die Empfehlung zum Einsatz von ICI in Kombination mit Chemotherapie. Bei einem CPS ≥ 10 wird Pembrolizumab (Keynote 590 [37]) und bei einem TC/TPS ≥ 1 % Nivolumab oder die Kombination aus Ipilimumab (Anti-CTLA-4-Antikörper) und Nivolumab empfohlen (CheckMate 648 [8]). In den weiteren Therapielinien findet auch der PD-1-Inhibitor Tislelizumab Anwendung (RATIONALE 302 [35]). Zudem besteht die Option einer weiteren antikörpervermittelten Mono- oder Kombinationstherapie durch Blockade des vaskulären endothelialen Wachstumsfaktorrezeptors 2 (VEGFR-2) durch Ramucirumab [41].

### Hepatobiliäre Tumoren

Die Therapie des fortgeschrittenen hepatozellulären Karzinoms (HCC) war lange Zeit auf den Tyrosinkinaseinhibitor Sorafenib beschränkt. Die Immuntherapie brachte für das HCC durch den ICI Atezolizumab (Anti-PD-L1-Antikörper) in Kombination mit dem Anti-VEGF-Antikörper Bevacizumab eine neue Option, die sich zum Standard entwickelte ([24]; IMbrave150; [10]). Es folgte die Zulassung der ICI-Kombination aus Tremelimumab (Anti-CTLA-4-Antikörper) mit Durvalumab (HIMALYA; [1]). Die Kombination der beiden Antikörper Nivolumab und Ipilimumab ist im HCC ebenso wirksam und wurde im Frühjahr 2025 zugelassen (CheckMate 9DW; [11]). Der Einsatz in weniger fortgeschrittenen Stadien der Erkrankung, die Kombination mit lokoregionären Verfahren, wie transarterieller Chemoembolisation (TACE) oder Radiofrequenzablation (RFA), sowie die beste Sequenz der zugelassenen Therapieoptionen ist dabei Teil intensiver Forschung. Die Kombinationstherapie aus Tremelimumab und Durvalumab nach lokoregionärer Therapie zeigte Effektivität beim HCC wobei Nichtansprechen mit einer starken Interaktion von T_reg_-Zellen mit CD8-positiven T‑Zellen in der Tumormikroumgebung einherging [25].

Beim intra- und extrahepatischen cholangiozellulären Karzinom (CCC) sowie Gallenblasenkarzinom hat die Immuntherapie ebenso Einzug gefunden. Im fortgeschrittenen Stadium ohne Option einer Resektion wird in der Erstlinientherapie aufgrund der Daten der TOPAZ-1 Studie eine Kombinationstherapie aus Gemcitabin und Cisplatin mit dem ICI Durvalumab eingesetzt [26]. Alternativ kann die Chemotherapie mit Pembrolizumab ergänzt werden analog der KEYNOTE-966 Studie [16].

### Kolon- und Rektumkarzinom

Das Kolonkarzinom stellt ein Beispiel für die Wirksamkeit von Immuntherapie aufgrund molekularer Eigenschaften des Tumors und deren Auswirkungen auf das Tumormikromilieu dar [29]. Der Nachweis eines MSI-Status im Kolonkarzinom ist dabei wegweisend für den Einsatz einer Immuntherapie. Im metastasierten nichtresektablen Stadium wird daher bei MSI-high- bzw. dMMR-Status der Einsatz von Pembrolizumab empfohlen. Dies basiert u. a. auf der Keynote-177-Studie und gilt ebenso für das metastasierte nichtresektable Rektumkarzinom [4]. Sollte bereits eine Chemotherapie erfolgt sein, kann bei MSI-high/dMMR-Status auch in der späteren Therapielinie eine Immuntherapie mit Nivolumab und Ipilimumab im kolorektalen Karzinom erfolgen (CheckMate 142 [18]). Diese Kombination erbrachte in der CheckMate-8HW-Studie bei MSI-high/dMMR-Tumoren auch im direkten Vergleich mit Chemotherapie einen Überlebensvorteil in der Erstlinientherapie, sodass die Erweiterung um diese Option im Frühjahr 2025 erfolgte [3].

Bei MSI-high- bzw. dMMR-Status wird der Einsatz von Pembrolizumab oder der Kombination Nivolumab/Ipilimumab empfohlen

Für die neoadjuvante Therapie zeigte sich für die Kombination aus Nivolumab und Ipilimumab im lokal fortgeschrittenen dMMR-Kolonkarzinom in der NICH-2-Phase-II-Studie in 98 % der Fälle ein pathologisches Ansprechen und in 68 % der Fälle eine Komplettremission [7].

Wegweisende Daten erbrachte die Therapie mit dem Anti-PD-1-Antikörper Dostarlimab in Patienten mit MSI-high-Rektumkarzinom im lokalisierten Stadium. Hier wurde in einer Phase-II-Studie mit 12 Patienten eine komplette Remission nach 6 Monaten bei allen Patienten erzielt [6].

Zusammenfassend sind aktuell führend ICI- und antikörpervermittelte Strategien Teil der Standardtherapie bei GI-Tumoren. Das Immunkonjugat Trastuzumab-Deruxtecan ist dabei ein Beispiel für die Weiterentwicklung dieser Therapieform. Die immunhistochemische Auswertung der Expression von Checkpointmolekülen hat sich dabei als guter Biomarker für den Einsatz von ICI, zumindest in manchen Tumorentitäten, erwiesen.

Im Verlauf soll daher auf vielversprechende Erkenntnisse und Entwicklungen eingegangen werden, die das Portfolio an Immuntherapien für GI-Tumoren in Zukunft erweitern könnten, um so Wirksamkeit und Ansprechraten zu verbessern.

## Der Blick in die Glaskugel

### Weitere antikörpervermittelte Immuntherapien

Die Erfahrung mit antikörpervermittelten Immuntherapien ist mittlerweile groß. Dennoch sind die Ansprechraten weiterhin niedrig, sodass sich die aktuelle Forschung auf neue Zielstrukturen, wie TIGIT, LAG3 und „T cell immunoglobulin and mucin domain-3“ (TIM-3), sowie die Optimierung der Antikörperstruktur konzentriert [21].

Der TIGIT wird auf Tumorzellen und aktivierten T‑Zellen exprimiert und in der fortgeschrittenen Phase-III-Studie SKYSCRAPER-07 für das Plattenepithelkarzinom des Ösophagus evaluiert [12]. Weitere Studien mit solide Tumoren im metastasierten Stadium untersuchen die Kombination einer Anti-TIGIT-Therapie mit ICI und Chemotherapie [32]. In präklinischen Modellen des nichtkleinzelligen Lungenkarzinoms wurde gezeigt, dass die Anti-TIGIT-Therapie die Antigenpräsentation auf Makrophagen stimuliert und eine Aktivierung von CD8-positiven T‑Zellen bedingt, was mit einer besseren Tumorkontrolle einherging.

Ein weiterer Immuncheckpoint ist LAG3, der intensiv erforscht wird. Die genaue Rolle von LAG3 in der Tumormikroumgebung stellt sich heterogen dar und hängt unterschiedlich von der Tumorentitäten und jeweiligen exprimierenden Zelle ab. Die Expression von LAG3 wird auch als prognostischer Faktor intensiv untersucht. Eine höhere Expression von LAG3 im Magenkarzinom war z. B. mit einer besseren Prognose assoziiert [39]. Die Kombination aus einem Anti-LAG3- mit einem Anti-PD-1-Antikörper ist im metastasierten Melanom bereits zugelassen, klinische Studien in GI-Tumoren werden durchgeführt [2].

Eine höhere Expression von LAG3 im Magenkarzinom war mit einer besseren Prognose assoziiert

Die TIM‑3 ist ein Immuncheckpointmolekül, dessen Blockade bereits in klinischen Studien Anwendung findet. Die Expression von TIM‑3 könnte auch als Biomarker genutzt werden [33]. Die Monotherapie mit Anti-TIM-3-Antikörpern zeigt jedoch nur eine geringe antitumorale Wirkung. Daher nutzen aktuelle Therapiestrategien die Kombination aus Anti-PD-1-/-PD-L1-Antikörpern. Bei Resistenz gegen Anti-PD-1/PD-L1-Antikörpern konnte eine Hochregulierung von TIM‑3 beobachtet werden [45]. Bei teilweise ernüchternden Ergebnissen bleibt die weitere Entwicklung abzuwarten.

Die Herstellung von bi- oder trispezifischen Antikörpern ist möglich, sodass 2 oder 3 Zielmoleküle simultan blockiert werden können [44]. Dabei werden Veränderungen der Bindungsregion genutzt, um die Wirkung von ICI gegen bekannte Moleküle wie CTLA‑4 weiter zu optimieren. Die Kombination aus dem modifizierten Anti-CTLA-4-Antikörper Botensilimab mit dem Anti-PD-1-Antikörper Balstilimab zeigte in einer Phase-I-Studie eine ermutigende Ansprech- und Erkrankungskontrollrate in der für die Immuntherapie schwer zugänglichen Subgruppe der MSS-Kolonkarzinome [5].

### Zelluläre Therapien

Zelluläre Therapien sind bei hämatologisch-onkologischen Erkrankungen bereits Standard, während sich der Einsatz bei soliden Tumoren aufgrund des immunsuppressiven Tumormikromilieus sowie der unzureichenden Persistenz der infundierten Zellen als schwierig erweist.

Der adoptive Zelltransfer von aktivierten T‑Lymphozyten wird intensiv erforscht, besonders die Nutzung neoantigenspezifischer T Zellen [27]. Die CAR-T-Zellen sind modifizierte T‑Zellen, die eine möglichst spezifische und starke zytotoxische Reaktion gegen Tumoren auslösen sollen. In soliden Tumoren werden spezifische Zielstrukturen, wie Claudin-18.2 oder Claudin‑6, zur Herstellung von CAR-T-Zellen genutzt, die unter anderem in Kombination mit immunstimulierender RNA eingesetzt werden [22, 28]. Ein weiterer Ansatz ist Glypican 3 (GPC3), das vermehrt auf HCC-Zellen exprimiert wird. In Mausmodellen zeigten GPC3-spezifische CAR-T-Zellen eine gute Wirksamkeit gegen HCC-Tumoren und werden aktuell in einer Phase-I-Studie überprüft [43]. Um die Persistenz zu verbessern, wurden GPC3-CAR-T-Zellen entwickelt, die zusätzlich Interleukin(IL)-15 exprimieren und dadurch eine bessere Expansion und Aktivität im Tumor zeigen [36].

### Onkolytische Viren

Onkolytische Viren sind bereits Teil der Therapieleitlinien im malignen Melanom. Die GI-Tumoren stellen jedoch weiterhin eine Herausforderung für diese Form der Immuntherapie dar. Dabei bestehen ähnliche bereits diskutierte Probleme wie fehlende Persistenz in der immunsuppressiven Tumormikroumgebung, fehlende Effektivität und unzureichende Immunogenität. Onkolytische Viren stellen dabei jedoch eine gute Plattform für die genetische Manipulation dar, wodurch weitere Moleküle wie z. B. Zytokinimmunkomplexe encodiert, durch das Virus transportiert und so vergleichsweise gezielt zur Tumorzelle gebracht werden können. Onkolytische Viren werden dabei insbesondere genutzt, um ansonsten immunogen „kalte“ Tumoren für eine ICI-Therapie zugänglich zu machen [13, 19].

### Therapieansätze bei problematischen Subgruppen

Immunsuppressive Tumormikroumgebungen und immunogen „kalte“ Tumoren sind weiterhin eine Herausforderung für die Immuntherapie. Die Euphorie über die Ansprechraten bei MSI-high/dMMR-Tumor kann nicht auf MSI-low/pMMR-Tumoren übertragen werden, da diese Subgruppe weiterhin immuntherapeutisch schwer therapierbar ist. Tumoren mit dichtem fibrösem Stroma, wie z. B. das Pankreaskarzinom, sind für zytotoxische Immunzellen schwer zugänglich und daher ist die Nutzung von Immuntherapeutika eingeschränkt. Ein vielversprechender Ansatz im Pankreaskarzinom kombiniert eine personalisierte mRNA-Neoantigen-Vakzine mit ICI sowie Chemotherapie, was mit einem besseren rezidivfreien Überleben einherging [31].

### Neue praxistaugliche Biomarker

Eine Herausforderung ist die Entwicklung praxistauglicher Biomarker. Aktuelle Biomarker basieren auf immunhistochemischen (CPS, TPS) oder genetischen Analysen (MSI/MSS). Komplizierte Berechnungen aus z. B. Einzelzellanalysen oder genetischen Expressionsprofilen scheinen dabei gute prädiktive Werte zu liefern, sind für den klinischen Alltag jedoch meist nicht umsetzbar. Der Einsatz von künstlicher Intelligenz zur Bildanalyse und Berechnung könnte dabei einen entscheidenden Fortschritt liefern [9].

Der Blick in die Glaskugeln zeigt, dass es nicht an neuen immuntherapeutischen Strategien mangelt. Da wahrscheinlich sowohl die kurzfristige Entwicklung neue Optionen für den klinischen Alltag erbringen als auch mittel- und langfristig weitere Entitäten und frühere Stadien erschlossen werden, lohnt es das Feld der Immuntherapie für GI-Tumoren engmaschig zu verfolgen.

## Fazit für die Praxis

- ICIs sind Teil der leitliniengestützten Standardtherapie von GI-Tumoren.
- Tumore mit Mikrosatelliteninstabilität (MSI)/defizientem Mismatch-Reparatur-System (dMMR) stellen eine Sondersituation für den Einsatz der Immuntherapie dar und gehen mit überdurchschnittlich gutem Ansprechen einher.
- Die klinische Entwicklung ist rasant und neue Therapiestrategien sind an der Schwelle zur Zulassung.
- Neue Therapiestrategien beinhalten neue Zielstrukturen für die antikörperbasierte Therapie, zelluläre Therapie mittels adoptivem Transfer, T‑Zellen mit chimärem Antigenrezeptor (CAR), onkolytische Viren und die Kombination verschiedener Ansätze.
- Kombinationen verschiedener Antikörper zur simultanen Bindung unterschiedlicher Immuncheckpointmoleküle sowie die Bindung von Antikörpern an andere zytotoxische Moleküle (Immunkonjugate) sind möglich.
- Durch die gleichzeitige Expression von Zytokinen oder (Neo‑)Antigenen können bisher wenig wirksame Immuntherapien effektiver werden.
- Tumore mit geringer Immunogenität, wie Pankreaskarzinom und MSS-Kolonkarzinom, stellen eine besondere Herausforderung dar.
- Biomarker zur Vorhersage des Ansprechens sowie der Effektivität sind limitiert und müssen mitentwickelt werden.
